# Reliable direct measurement of causes of death in low- and middle-income countries

**DOI:** 10.1186/1741-7015-12-19

**Published:** 2014-02-04

**Authors:** Prabhat Jha

**Affiliations:** 1 Centre for Global Health Research (CGHR), St. Michael’s Hospital and Dalla Lana School of Public Health, University of Toronto, Toronto M5B 1C5, Canada

**Keywords:** Causes of death, Cause of death statistics, Mortality, Vital statistics, Verbal autopsy, Sample registration system

## Abstract

**Background:**

Most of the 48 million annual deaths in low- and middle-income countries (LMICs) occur without medical attention at the time of death so that the causes of death (COD) are largely unknown. A review of low-cost methods of obtaining nationally representative COD data is timely.

**Discussion:**

Despite clear historic evidence of their usefulness, most LMICs lack reliable nationally representative COD data. Indirect methods to estimate COD for most countries are inadequate, mainly because they currently rely on an average ratio of 1 nationally representative COD to every 850 estimated deaths in order to measure the cause of 25 million deaths across 110 LMICs. Direct measurement of COD is far more reliable and relevant for country priorities. Five feasible methods to expand COD data are: sample registration systems (which form the basis for the ongoing Million Death Study in India; MDS); strengthening the INDEPTH network of 42 demographic surveillance sites; adding retrospective COD surveys to the demographic household and health surveys in 90 countries; post-census retrospective mortality surveys; and for smaller countries, systematic assembly of health records. Lessons learned from the MDS, especially on low-cost, high-quality methods of verbal autopsy, paired with emerging use of electronic data capture and other innovations, can make COD systems low-cost and relevant for a wide range of childhood and adult conditions.

**Summary:**

Low-cost systems to obtain and report CODs are possible. If implemented widely, COD systems could identify disease control priorities, help detect emerging epidemics, enable evaluation of disease control programs, advance indirect methods, and improve the accountability for expenditures of disease control programs.

## Background

Reliable, reproducible, and openly available information on age-specific and sex-specific cause of death (COD) is essential to charting pathways to reduce premature child and adult mortality [1-5]. The United Nations (UN) estimates that the 48 million deaths in low- and middle-income countries (LMICs), including about 7 million child deaths, represent 86% of the 56 million global deaths that occurred in 2010 [6]. Most deaths in LMICs do not have a diagnosis of COD [3,7]. Given the limitations of indirect methods to estimate COD statistics, the most urgent need is to collect reliable, representative, and robust primary COD data to guide national and global health action and research. There are practical ways for LMICs to develop and expand rapidly the mortality systems that determine the CODs. This paper draws on the lessons from various countries, in particular from the ongoing Indian Million Death Study (MDS) [8,9].

### Mortality and causes of death

There are two components to measuring mortality; the first is capturing the event of death (by at least age and sex, but ideally including geographical residence and education or some measure of social status). The second is ascertaining the underlying COD, which is the disease that contributed most directly to the death (as distinct from immediate cause or co-existing conditions)*.* Death is a concrete, final, and measurable event that households remember well, and it can be captured in household surveys reliably.

Since about 1960, use of population totals from census data and retrospective mortality estimates, paired with indirect demographic methods, has enabled reasonably credible estimates of the age-specific and sex-specific national mortality rates for most countries. These are published and updated regularly by the UN Population Division [6]. Additional methods such as household surveys of birth and death histories of children, their siblings and parents, or similar methods [4,10,11], have also improved estimates of age- and sex-specific mortality, or in some cases specific causes (such as maternal deaths).

Even rudimentary data on age- and sex-specific all-cause mortality rates can identify trends for rapidly changing diseases such as those responsible for childhood mortality [12]. A simple analysis of the excess death rates between the 1911 and 1921 censuses in India suggests that the influenza epidemic of 1918/19 might have killed about 20 million of the population [13]. More recently, it has been possible to obtain a crude estimate of the effect of HIV infection on mortality in selected sub-Saharan countries from the observed large increases in all-cause mortality rates in young adults from 1990 to 2000, using simple burial records [14]. In both cases, this was because the ‘signal’ of influenza-attributable or HIV-attributable mortality (the increase in the number of deaths) exceeded the ‘noise’ of misclassification by competing mortality from other causes [15].

### Why collect cause of death data?

Over the past century, reliable COD data in high-income countries have led to numerous discoveries and health improvements [1,16-18]. For example, the dramatic increase in lung cancer deaths in British and American men around the period of World War II spurred research that led to the identification of smoking as a cause of lung cancer and, eventually, a wide range of diseases [19]. In the early 1980s, routine mortality data from San Francisco identified an exceptional increase in immune-related deaths in young men, which signaled the beginning of the American HIV epidemic [20]. Downward trends in infectious disease mortality worldwide have reflected the widespread use of immunizations and antimicrobials [17,18].

**Figure 1:**
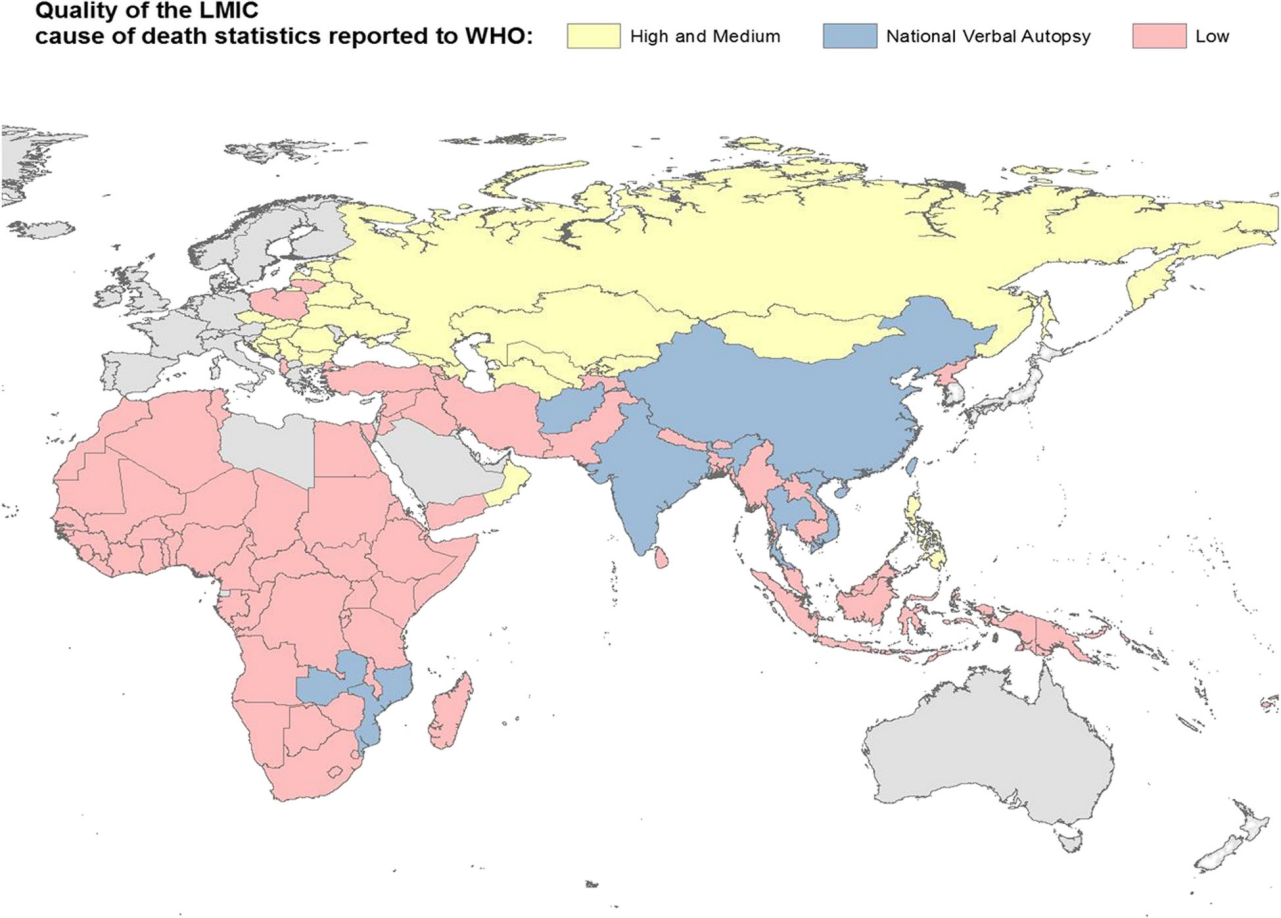
**Quality of the cause of death statistics from low- and middle-income countries (LMICs) reported to WHO.** Only five countries outside China, India and Latin America have national COD surveys: Afghanistan, Mozambique, Thailand, Vietnam and Zambia. Zambia's survey covers 4 of 10 provinces. The remaining countries are nationally representative. Source: Mathers *et al*. [3], updated based on data from Murray *et al*. [27].

Despite these clear benefits, COD data are exceptionally limited in LMICs because of historical neglect and insufficient resources. Of 115 countries that reported mortality data to the World Health Organization (WHO) around 2005, only 64 had good-quality vital registration that also included COD certification [3]. Fully 75 countries (including about 90% of African countries) did not provide data on COD for any year after 1990 (Figure 1). Similarly, the Child Health Epidemiology Reference Group estimated that COD information was available for only 3% of global child deaths [7]. There is a major knowledge gap for representative information on COD in adults, such as the proportion of adult deaths due to malaria [21]. China and India have nationally representative COD surveys [9,22,23], and China and Latin America have expanded civil registration with medical certification. Thus, the major gap in COD information exists in sub-Saharan Africa and in South and East Asian countries [3].

### Seven limitations of indirect methods to estimate COD

In 1981, the Ghana Health Assessment Team [24] advanced the concept of measuring burden of disease by combining loss of useful life through premature death with the loss of useful life due to disability. This spurred further work, most notably the influential World Development Report 1993 by the World Bank [25]. The WHO Global Health Estimates (GHE) [26] use similar approaches to estimate mortality levels and COD for the major regions of the world, and for most countries. The 2010 Global Burden of Disease (GBD) Study goes further by claiming it can make reliable estimates for ‘291 diseases and injuries, 67 risk factors, and 1,160 non-fatal health consequences in 21 regions, 20 age groups and 187 countries’ [27]. It is important to understand the strengths and limitations of these indirect methods.

**Figure 2:**
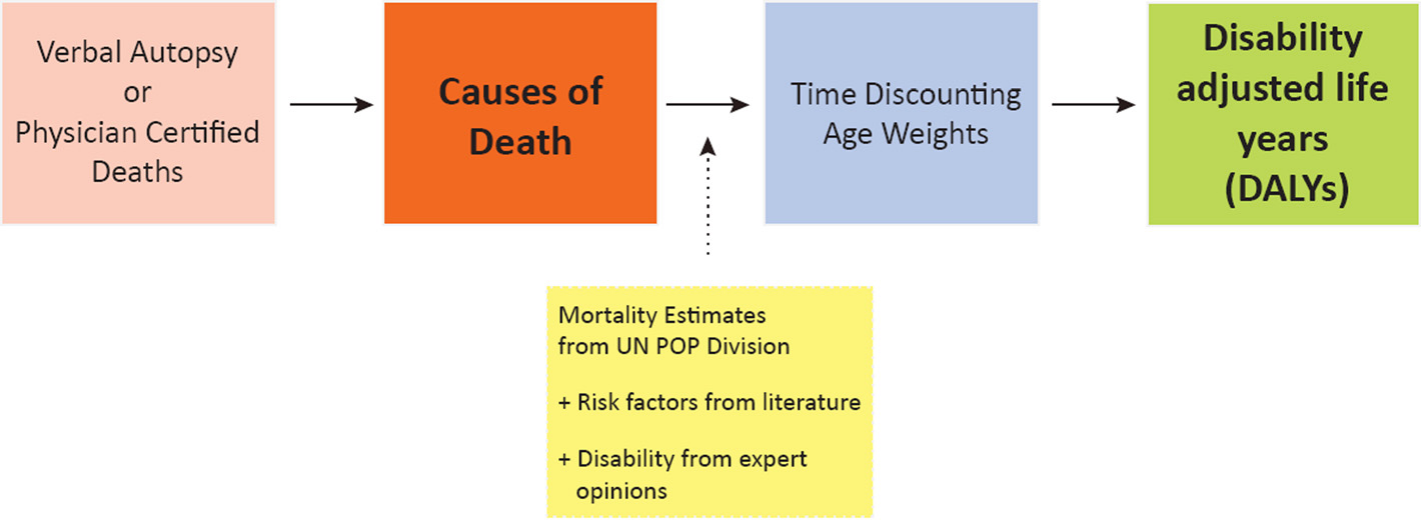
**Simplified schema of death and disability estimates in indirect methods.** Modified from World Bank data [25].

A simplified schema of how GBD estimates are created is shown in Figure 2, with the key inputs of mortality totals (derived from the UN or demographic methods), COD data, estimates of disability weights for specific conditions (based largely on expert opinion), and risk factors (based largely on literature reviews). The GBD uses regression equations (of uncertain error) to predict the level of mortality, or the distribution of several CODs from populations (mostly in high-income countries) for which such data are known. Parameters for independent variables include economic (GDP/capita), educational (literacy rates), and other variables such as generalized HIV epidemic, endemic malaria, and geography, among others. In parallel, simultaneous equations are used to model relations between incidence, prevalence, death rate, duration of disease, and survival, among others, although this does not directly enable estimations of COD distributions.

**Figure 3:**
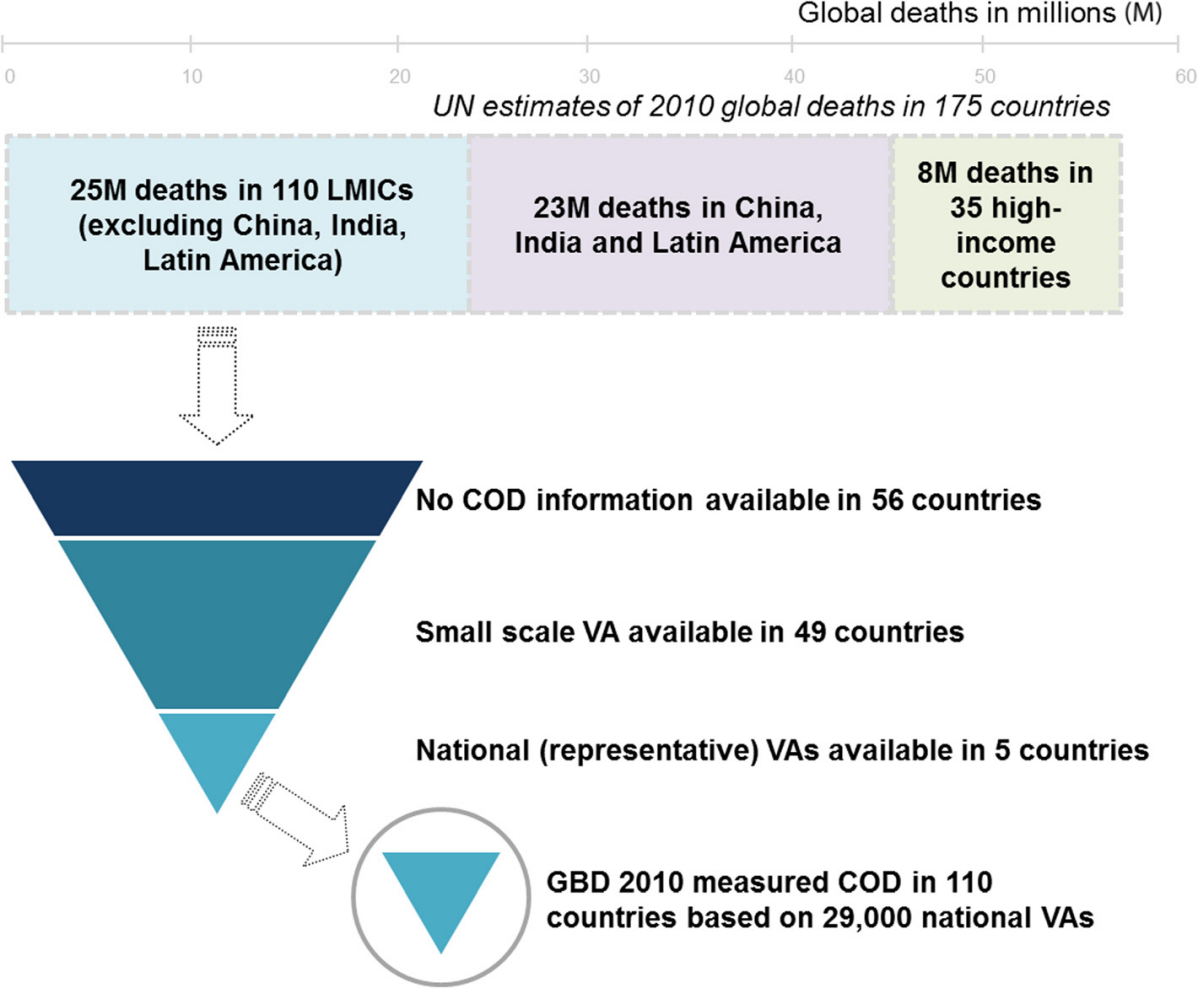
**Cause of death (COD) data in the Global Burden of Disease (GBD) study for selected countries.** The GBD measured the major COD types in 110 low- and middle-income countries (LMICs) based on data available from national surveys with verbal autopsy (VA) in five countries; final results were based on a ratio of estimated to actual COD data that was less than 0.12% (or about 1 in 850). Most health measurements rely on disability estimates as opposed to mortality, and measurement error often exceeds the desired change in health outcomes following an intervention. For example, in seeking a 10% improvement in health outcomes in children under 5 years of age, it is not possible to accurately assess the outcome of an intervention if the measurement error exceeds 10%. By contrast, as death is a discernible, objective outcome, restricting analyses to mortality significantly reduces measurement error [1,28].

The obvious limitation of this indirect method is an insufficient number of nationally representative COD data. The 2010 GBD [27] study had to apply these complex methods to a ratio of 1 nationally representative COD to 850 estimated deaths so as to measure the cause of 25 million deaths, across 110 LMICs outside China, India, and Latin America (Figure 3). National COD surveys of reasonable representativeness used by the GBD were available only from Afghanistan, India, Mozambique, Thailand, Vietnam, and Zambia, totaling about 60,000 surveyed annual deaths [27], of which half were from the MDS in India [9]. Other COD data sources in the GBD are described scantily, but appear to comprise hospital archives on deaths and diseases (a patchwork of generally small, non-representative surveys), death and cancer registries, mortuary statistics, and incomplete records from ministries, census departments, and police reports.

Aside from this key data limitation, the GBD has six other constraints, as follows:

1) The core COD data are not open source, and it has not thus far been possible to reproduce GBD results, limiting scientific confidence in its findings [2,29-32].

2) Most econometric models have little ability to deal with true underlying uncertainty, as they have few robust COD data sources available, and most importantly, have too few representative COD surveys. This in turn creates large variations in individual diseases from unstable models that are not specific to local country conditions [33]. For instance, the 1996 version of the GBD estimated 0.95 million deaths from tuberculosis in India for the year 2000, whereas the 1999 version estimated 0.42 million deaths for the year 1998 [32]. Similarly, recent estimates of childhood and adult deaths from malaria in Africa [34] have partially modeled the Indian MDS mortality patterns [21] onto the COD distribution in Africa. The net result may be an underestimation of the extent to which malaria in Africa kills children, and an overestimation of the extent to which it kills adults. Similarly, changes in the reporting of maternal deaths mean that the GBD estimates of maternal deaths due to human immunodeficiency virus (HIV) infection vary from 18,000 to 56,000 in adjacent years [35]. Finally, the use of economic and educational variables in regression equations might falsely reduce outliers for countries that produce better mortality outcomes at lower spending (or *vice versa)*.

3) The models do not adequately capture the underlying uncertainty in misclassification of CODs, but rather create somewhat misleading ‘uncertainty intervals’ based on re-running the econometric simulations. Thus, the GBD has equally narrow estimates for the range of diabetes deaths in Africa as for those in high-income countries, despite the obvious paucity in Africa of COD data or surveys of diabetes prevalence [31].

4) The GBD often rejects real epidemiological data if they do not fit the models [29,36]. Among Indian women aged 30 to 69 years, the MDS estimates that 33,000 die from cervical cancer and 20,000 from breast cancer [37], whereas the GBD claims that more Indian women die from breast than from cervical cancer.

5) The total numbers of deaths in the GBD are uncertain as these too have relied on regression models for estimation of total mortality. The GBD estimates nearly 3 million fewer deaths in LMICs than does the UN Population Division for 2010 [6]. The UN and the South African Medical Research Council estimated a 3% yearly decrease in childhood mortality in South Africa since 2000, mostly based on direct surveys, whereas the GBD estimates projected a 3% yearly increase over the same time period [38]; obviously, both cannot be right.

6) Such indirect estimates, especially when published by international organizations in major journals, create a false impression that reliable country-level estimates are available, and this may inhibit further work to obtain direct estimates.

Mortality comprises most of the composite of mortality and disability measures, such as disability-adjusted life years [25]. Mortality does not capture all illnesses or specific priorities, such as depression, schizophrenia, or blindness. For most diseases, the GBD estimates of disability are largely expert opinion, and thus there is no easy way to validate the disability estimates or to study the relationship of disability and death. For example, in comparison to the 1993 World Bank report [25] or earlier GBD reports, the 2010 version of the GBD assigns about 2.5 times as many disability adjusted life years to child deaths [39]. However, the correlation between premature mortality (specifically death before the age of 70 years) and morbidity is strong for the majority of other diseases of public health importance, with only some exceptions [27,28]. This implies that reliable COD data should remain the major priority for most LMICs.

### Five options to expand COD reporting at low cost and high impact

For most individual LMICs, the sources of mortality data are too incomplete, too unrepresentative and of too uncertain quality for accurate COD estimation at the national level. Expanding the number of countries with direct COD data is also the best way to improve indirect estimates, and more importantly, to assist national public health action and research [1-5,28].

The major objective for most LMICs should be to obtain representative data on age-, gender-, and social strata-specific CODs (both total numbers and rates per living population) for the major diseases. Universal civil registration of deaths with medical certification remains the best long-term goal [11], but complete civil registration is achieved over several decades. Even the USA took the better part of a century to increase death certification, and some states did not reach complete coverage until the 1970s [1,16]. Developing countries have shown little progress in the expansion of civil registration [11], and COD statistics lag even further behind [4]. Slow progress in civil registration in LMICs is mostly a result of limited access to medical care and the fact that most deaths occur at home rather than in hospitals. Incentives for households to register deaths are limited, because pension and insurance schemes or enforceable familial inheritance and property rights are uncommon in LMICs. Strategies to increase civil registration include increasing medical attention at death and training existing healthcare workers to complete death certificates [11]. Requiring burial or cremation grounds to register deaths is practicable in urban settings, but currently less so in rural areas. Efforts to accelerate COD statistics can strengthen civil registration if they work in concert with national census and statistical organizations [4,9,11].

There are at least five complementary and practicable approaches to obtain better COD data in the medium term (such as by 2025) [1-5].

The first and most robust option (sometimes called Sample Vital Registration with Verbal Autopsy [8,9]) is the establishment of a Sample Registration System (SRS). Such a system has worked in representative areas of India since 1971 [9]. China has a similar but less representative system [23]. SRSs are able to continuously and prospectively collect data on the event of death and COD from enrolled households, enabling reliable determination of mortality rates in the population. Mortality rates may require adjustment, as in India, for undercounts that arise from loss to follow-up [40]. SRSs have the disadvantage of requiring more technical capacity to enable continuous enumeration compared with retrospective household surveys.

**Figure 4:**
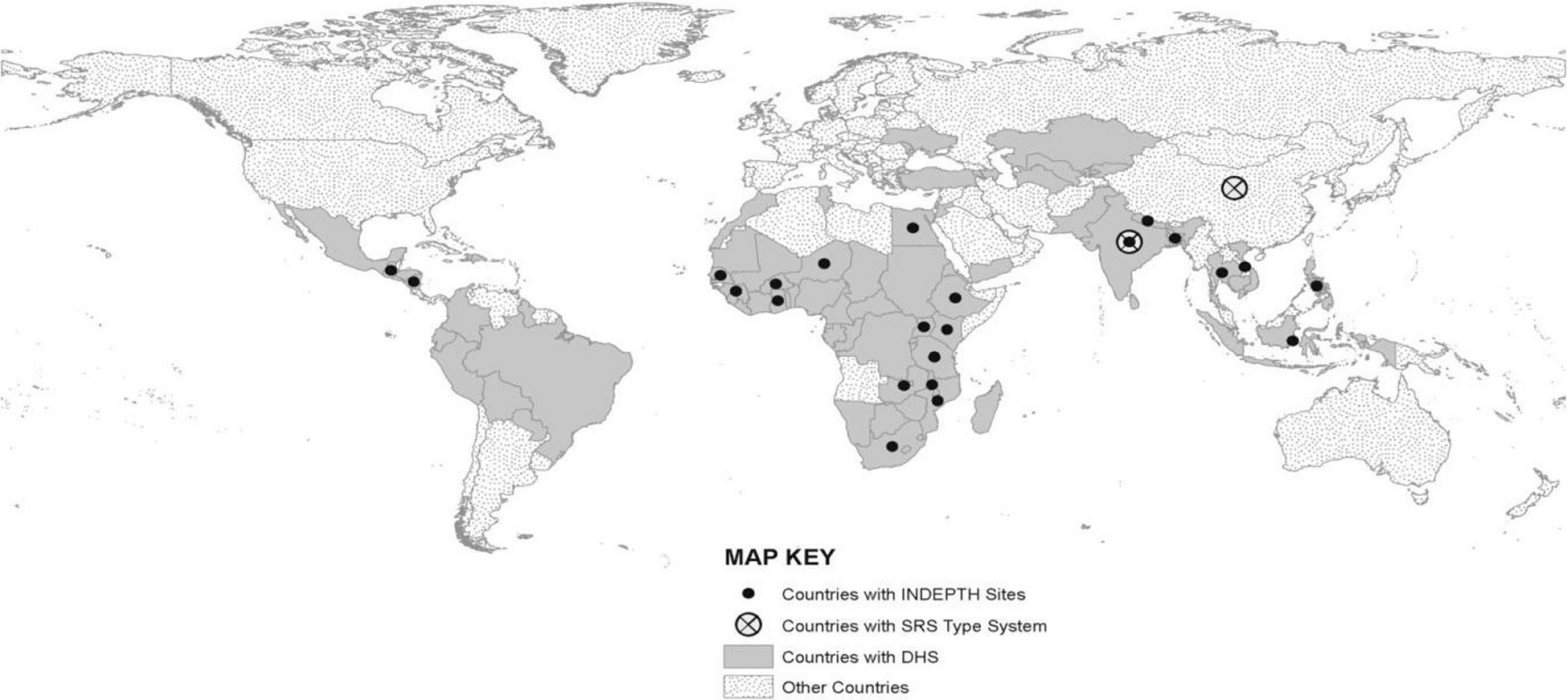
**Low- and middle-income countries with Demographic and Health Surveys (DHS), INDEPTH network Demographic Surveillance Sites, or Sample Registration Systems (China and India).** Source: INDEPTH [41] and DHS [43], reproduced with permission [1].

The second option is to strengthen the ongoing INDEPTH network [41,42] of about 42 demographic surveillance sites in 19 countries (Figure 4). This would involve efforts to expand the sample size in each country and make the sites representative of the population, and to enable prospective collection of deaths. The Adult Morbidity and Mortality Project in Tanzania was able to strengthen vital statistics and survey capacity, and enabled the use of COD data to monitor and evaluate malaria, HIV/AIDS, and other national programs [42].

The third option is to carry out retrospective surveys of CODs to supplement the globally standardized Demographic and Health Surveys (DHS). These surveys were initiated by the US government about three decades ago, and now cover about 90 countries (Figure 4, [43]). The addition of COD information for children to selected DHS has reduced much of the substantial uncertainty that existed in the 1960s [7]. DHS sample frames could be used to conduct follow-up retrospective COD surveys for children and adults.

The fourth option involves carrying out large, well-planned retrospective surveys of mortality using census sample frames. The marked variation in disease in China became apparent about 3 decades ago, following a complete national survey of the causes of 20 million deaths, carried out in the period 1973 to 1975 [44]. This survey yielded the first assessment of age-specific and cause-specific mortality rates for each province and county, and for China as a whole. The study showed a large variation in disease rates across the country, which in turn brought about health interventions and further research. A similar survey covered about 1 million deaths in 24 urban and 74 rural areas of China during 1986 to 1998 [45]. More recently, Mozambique conducted a national COD survey of over 11,000 deaths, based on deaths identified in the preceding census [46].

Finally, in small countries, health departments may record significantly more deaths and COD data (through hospitals and community health centers) than is recorded through civil vital registration, as has been documented recently for Tonga and Fiji [47-49]. Similarly, a small number of countries which have reasonable levels of civil registration of death but more limited COD data, should consider expanding medically certified COD while concurrently instituting household-based verbal autopsies (VAs) for deaths that occur without medical attention [1,4].

### Lessons learned from the Million Death Study in India

Various countries, notably China and India, have introduced large-scale VA studies [9,23]. The common lesson from these is the usefulness of even crude COD data that report on the most important diseases of public health importance. The MDS in India is a recent and one of the largest of these VA studies, so lessons from it are particularly relevant for other countries.

In the absence of reliable COD data, nationally representative VA surveys are a viable alternative. VAs involve a structured investigation of the circumstances and symptoms leading to a death, through interviewing an associate of the deceased during a household survey. VAs have inherent limitations, particularly the ability to identify only broad categories of diseases, and a high proportion of undetermined (or ‘ill-defined’) causes in people aged over 70 years [8,50]. Even in high-income countries, COD at older ages is difficult to document [51]. However, because the mean age of death in most LMICs is far lower than in developed countries, VA remains very relevant for public health and disease control priorities. With properly standardized training and data collection, double-coding by physicians, and other quality controls, the aggregation of VA data can be extremely powerful at the population level.

Prior to 2004, it was difficult in India to accurately assess local health conditions, explore key risk factors, and evaluate the effects of investments and policies. To address this fundamental gap, the MDS was launched in 2002 [8,9]. In collaboration with the Registrar General of India, the MDS applied simple, low-cost methods (less than US$2 per household) to record COD data from about 1 million homes throughout the country, which were randomly selected from the preceding census. The MDS focused on a simple process using local field staff who obtained key questions on all deaths, including a half-page narrative in the local language. These were converted into electronic records and coded independently by at least two physicians [8,9]. Household participation rates have averaged close to 100%. Despite the obvious limitations of VA, the COD information in India today is many times better than the little or no data available previously. Even crude COD data applied widely to various diseases and risk factors across an entire country have yielded unexpected results and influenced disease control priorities. Some selected results from the MDS and their implications for disease control are shown below:

• HIV/AIDS resulted in 0.1 million deaths in 2004 (UNAIDS estimated 0.4 million [52]). This result led to adjustments in AIDS funding to better align with the actual demand in India for life-prolonging therapies.

• Smoking caused approximately 1 million deaths in 2010 [53]. This finding supported the Indian government efforts to introduce warning labels on cigarette packages and to raise tobacco taxes to help reduce cigarette consumption.

• Malaria caused 0.2 million deaths (13 times the WHO estimate) in 2005, primarily in adults [21]. Within 1 week, this finding led to public demand for greater control of malaria in the state of Orissa (and spurred other, currently inconclusive, research on adult malaria deaths in Africa [34]).

• The survey identified 2 million child deaths in 2005 resulting from five avoidable causes [54]. This finding spurred the expansion of neonatal/intra-partum care and is presently enabling district-based monitoring of child deaths and up-to-date estimates of neonatal mortality by gender across all districts [55].

• Cervical cancer was the leading cause of cancer death in adult middle-aged women in 2010 [37], accounting for 33,000 deaths. If this corresponding rate were to be reduced to that seen in populations with low levels of transmission of the causative human papillomavirus, cervical cancer death rates in Indian women would be about two-thirds lower.

• Snakebite kills 50,000 people [56] in India, which equals the WHO estimate for all snakebite deaths worldwide. The distribution of snakebite deaths in specific states suggests that it might be possible to eliminate or substantially reduce deaths in these states.

The MDS has three key methodological implications relevant for other LMICs:

1) There is a need to avoid the false ‘gold standard’ of hospital CODs. Hospital-based deaths cannot be used to validate VA, because the age, educational level, pathogen distribution, and etiology of rural medically-unattended deaths differs hugely from hospital deaths [50,57]. Further studies are needed (and planned) to compare individual- and population-level results from VA collected by non-medical staff, but coded by a doctor, against deaths collected and certified by a doctor in rural areas. At the population level, simple but robust criteria to quantify the performance of VA-based COD systems are proposed and applied to the MDS [58].

2) The most practicable and robust measure to capture the true uncertainty in COD data is the proportion of ill-defined deaths before old age. It is simply impossible to know the causes for all deaths, especially those based on VA. Countries require a balance akin to St. Augustine’s plea ‘Oh Lord, let me be chaste, but not quite yet.’ Quality controls over fieldwork and coding can reduce ill-defined conditions without artificially eliminating them. Reductions in the proportion of these ill-defined conditions indicate improvements in quality, but no ill-defined conditions indicate fraud or deficiencies in fieldwork [58]. This further suggests a need to re-examine the GBD and WHO practice of re-assigning the less-defined codes in the International Classification of Diseases [59] (sometimes called ‘garbage codes’). Re-assignment may well muddy COD data by combining truly ill-defined causes with other, more certain, causes [28].

3) COD systems can also measure risk factors, in particular for adult CODs. For most infectious conditions, such as meningitis, the COD is directly related to the infective agent. By contrast, chronic diseases such as myocardial infarction may be caused by several factors such as smoking, raised blood pressure, or raised blood lipids. Co-morbidity codes on death certificates [60] have been used in high-income countries, but completion of these codes on death certificates in LMICs remains uncommon. Simply asking about the dead person’s risk factors can be useful. A retrospective study of one million deaths in China compared the proportions of smokers and non-smokers who died of tobacco-attributable diseases versus non-tobacco-related diseases (chiefly injuries) to calculate the excess number of deaths in smokers [45]. These methods have been extended recently to South Africa [61] and Bangladesh [62]. Simple questions in the MDS about the deceased’s risk factors and that of the respondent enabled household case–control methods to quantify tobacco smoking, tobacco chewing, and alcohol drinking hazards in India [53]. The household interviews required for VA enable the collection at low cost of a wider range of other exposures, and information on use of health and other social services.

Household visits are a unique opportunity to obtain representative information on how people live, and then to link these to deaths. Geocoding these data provides unprecedented capability to use geospatial epidemiology and data-mining approaches to understand disease and its determinants. Examples of information available from rapid, standardized interviews of the household include the following:

1) **Childhood** immunization, treatment-seeking, service use, nutrition patterns, and other variables.

2) **Maternal** contraception and birth-spacing patterns, and use of maternal services and newer technologies.

3) **Access** to and use of HIV, tuberculosis, malaria, and other services, including use of mosquito nets, condoms, and other preventive measures.

4) **Household** nutrition, financial protection, access to microcredit, and spending or borrowing for disease treatment.

5) Connectedness with **mobile technology**, modern banking, identity schemes, health insurance, and access to markets for agricultural or other goods made locally.

6) Simple dried blood spots or oral saliva samples to enable **‘bio-surveys**’ **or** ‘**sero-surveillance**’ of various infections, validate coverage rates against major antigens, determine prevalence of diseases such as malaria and dengue, and enable multiplex central testing [63] of various biological correlates of disease.

### Faster, cheaper, and better nationally representative COD surveys

Information technology, most notably electronic capture of field data with global positioning satellite tracking and electronic coding by physicians, biological co-sampling of deaths, and use of computer algorithms and geospatial sampling, could substantially improve the validity, reliability, and feasibility of VA, while simultaneously lowering costs. Computer-assisted coding is a promising complement to physician coding, which remains the preferred standard [64]. Future combinations of algorithms might well improve or, less certainly, even replace physician coding.

The large-scale demonstration of the technical and political feasibility of the MDS, paired with the aforementioned innovations in COD surveys, suggests that it is possible for LMICs to adopt at low cost a number of methods to obtain nationally representative COD data by age and gender. A key emerging theme is the need for all such data to be fully and openly available (with protection for individual data and privacy) to ensure that these may be used imaginatively and without restriction [65]. A reasonable goal would be to ensure that all LMICs, but particularly low-income African and Asian countries, have in place large, simple, nationally representative surveys of COD by 2025 [66,67] in time to monitor the proposed replacement for 2030 of the UN global development goals [39,68].

## Summary

Representative and accurate COD information systems have historically been of huge importance to public health. Such low-cost systems are possible, and if implemented widely, can identify disease control priorities, advance GBD, GHE and other indirect methods, help detect emerging epidemics, enable evaluation of disease control programs, and improve the accountability for expenditures of disease control programs [1-5,28].

## Abbreviations

COD: Cause of death; GBD: Global Burden of Disease; GHE: Global Health Estimates; LMIC: Low- and middle-income countries; MDS: Million Death Study; TB: Tuberculosis; VA: Verbal autopsy; WHO: World Health Organization.

## Competing interests

The author declares that he has no competing interests.
